# Biomechanics of the medial meniscus in the osteoarthritic knee joint

**DOI:** 10.7717/peerj.12509

**Published:** 2021-11-24

**Authors:** Karol Daszkiewicz, Piotr Łuczkiewicz

**Affiliations:** 1 Department of Mechanics of Materials and Structures, Faculty of Civil and Environmental Engineering, Gdańsk University of Technology, Gdańsk, Poland; 2 II Department of Orthopaedics and Kinetic Organ Traumatology, Medical University of Gdańsk, Gdańsk, Poland

**Keywords:** Knee osteoarthritis, Medial meniscus, Meniscal tear, Meniscal extrusion, Knee biomechanics, Hoop stress

## Abstract

**Background:**

Increased mechanical loading and pathological response of joint tissue to the abnormal mechanical stress can cause degradation of cartilage characteristic of knee osteoarthritis (OA). Despite osteoarthritis is risk factor for the development of meniscal lesions the mechanism of degenerative meniscal lesions is still unclear. Therefore, the aim of the study is to investigate the influence of medial compartment knee OA on the stress state and deformation of the medial meniscus.

**Methods:**

The finite element method was used to simulate the stance phase of the gait cycle. An intact knee model was prepared based on magnetic resonance scans of the left knee joint of a healthy volunteer. Degenerative changes in the medial knee OA model were simulated by nonuniform reduction in articular cartilage thickness in specific areas and by a decrease in the material parameters of cartilage and menisci. Two additional models were created to separately evaluate the effect of alterations in articular cartilage geometry and material parameters of the soft tissues on the results. A nonlinear dynamic analysis was performed for standardized knee loads applied to the tibia bone.

**Results:**

The maximum von Mises stress of 26.8 MPa was observed in the posterior part of the medial meniscus body in the OA model. The maximal hoop stress for the first peak of total force was 83% greater in the posterior horn and only 11% greater in the anterior horn of the medial meniscus in the OA model than in the intact model. The reduction in cartilage thickness caused an increase of 57% in medial translation of the medial meniscus body. A decrease in the compressive modulus of menisci resulted in a 2.5-fold greater reduction in the meniscal body width compared to the intact model.

**Conclusions:**

Higher hoop stress levels on the inner edge of the posterior part of the medial meniscus in the OA model than in the intact model are associated with a greater medial translation of the meniscus body and a greater reduction in its width. The considerable increase in hoop stresses shows that medial knee OA may contribute to the initiation of meniscal radial tears.

## Introduction

Menisci are fibrocartilaginous structures that play a very important role in the mechanical protection of knee cartilage (Walker & Erkman, 1975; Chang et al., 2011). They enhance congruity, distribute stress and stabilize the knee joint. Loss of meniscal function due to a tear or degeneration leads to an increase in peak contact pressure across the knee joint and a risk of cartilage loss adjacent to the meniscal tear (Baratz, Fu & Mengato, 1986). Increased mechanical loading and pathological response of joint tissue to the abnormal mechanical stress contribute to degradation of cartilage characteristic of knee osteoarthritis (OA) (Felson, 2013).

Knee OA may also lead to a meniscal tear due to weakening of meniscal structure (Englund, Guermazi & Lohmander, 2009). Degenerative meniscus lesions are typically asymptomatic and are often associated with preexisting knee osteoarthritis (Noble & Hamblen, 1975; Stoller et al., 1987; Hodler et al., 1992; Lange et al., 2007). The meniscal lesions were found in 70–90% of knees among patients with symptomatic knee OA (Kornaat et al., 2006). Despite osteoarthritis is risk factor for the development of meniscal lesions (Englund et al., 2011), the mechanism of the degenerative meniscus lesions is still unclear (Furumatsu et al., 2019). Clinical studies are not sufficient to determine the pathogenesis of this disease due to the multiplicity of the etiological factors.

The finite element method (FEM) serves as a useful tool for investigating the mechanical status of the knee joint in different pathological conditions (Kluess et al., 2009; Łuczkiewicz et al., 2016). Previous works (Peña et al., 2005; Zielinska & Haut Donahue, 2006; Mononen, Jurvelin & Korhonen, 2013; Shriram et al., 2019; Zhang et al., 2019; Li et al., 2020) have focused on the influence of degenerative meniscal tears and meniscectomies on knee biomechanics. These FEM simulations were based on the geometry of healthy volunteers’ knee joints without changes typical of osteoarthritis. Moreover, the material properties of the menisci and cartilage did not reflect the changes typical of osteoarthritis in the previous studies. For this reason, such models were not useful for assessing the influence of degenerative changes on the pathomechanism of degenerative meniscal injury. The process of cartilage degeneration and damage was simulated in recent works (Liukkonen et al., 2017; Miller & Krupenevich, 2020). Peters et al. (2018) suggested that computational models of the knee joint should be constructed using material parameters and geometry from cohorts with consistent disease state.

The goal of this study was to examine the influence of changes in geometry and material parameters characteristic of medial knee OA on the biomechanics of the medial meniscus.

## Materials & Methods

### Geometry

A model of the left knee joint was prepared on the basis of magnetic resonance imaging (MRI) of a healthy volunteer (female, 43-year-old, 1.75 m, 68 kg) in a full extension position. A 1.5 T MRI scanner (Magnetom Aera, Siemens, resolution of 0.28125 mm) was used to obtain the images of the knee in the axial plane (slice spacing = 0.7 mm) and the sagittal and coronal planes (slice spacing = 3.6 mm). Written informed consent was obtained from patient to use data from her medical records in research. The study was approved by Independent Bioethics Committee for Research at Medical University of Gdańsk, Poland. Then, the MRI scans were imported into Mimics (Materialise NV, Leuven, Belgium), where semi-automatic segmentation was performed by an experienced user and verified by a radiologist. The obtained three dimensional (3D) model of the knee joint geometry consisted of bones (femur, tibia, fibula), menisci and articular cartilage (femoral and tibial).

### Finite element models

An intact finite element (FE) model of the knee joint was developed in Abaqus 6.14-2 (Dassault Systemes Simulia Corp., Providence, RI, USA) based on the 3D geometry imported from Mimics. Ligaments were modelled using two node, 3D axial connector elements CONN3D2. The following ligaments were included in the FE model: anterior cruciate ligament (ACL), anterior intermeniscal ligament (AIML), anterolateral ligament (ALL), lateral collateral ligament (LCL), medial collateral ligament (MCL), posterior cruciate ligament (PCL) and posterior oblique ligament (POL). The attachment points of the specific ligament bundles were identified in the MRI images. The medial collateral ligament was divided into deep bundle (dMCL) and three superficial bundles: anterior, middle and posterior. Two portions of the dMCL were distinguished: meniscotibial ligament (MTL) connecting the medial meniscus to the tibia and meniscofemoral ligament (MFL) connecting the medial meniscus to the femur. The MTL, MFL and AIML ligaments were modelled by the six axial connector elements to minimize stress concentrations at the attachment points to the medial meniscus.

Three additional models were prepared to examine the influence of medial knee OA on the biomechanics of the medial meniscus. Model G was introduced to assess the effect of only altered geometry of the articular cartilage corresponding to KL score 3 and model M to evaluate the influence of only decrease in material parameters characteristic of medial knee OA. Degenerative changes in the OA model were simulated by both non-uniform reduction in cartilage thickness and reduction in material parameters of cartilage and menisci.

**Figure 1:**
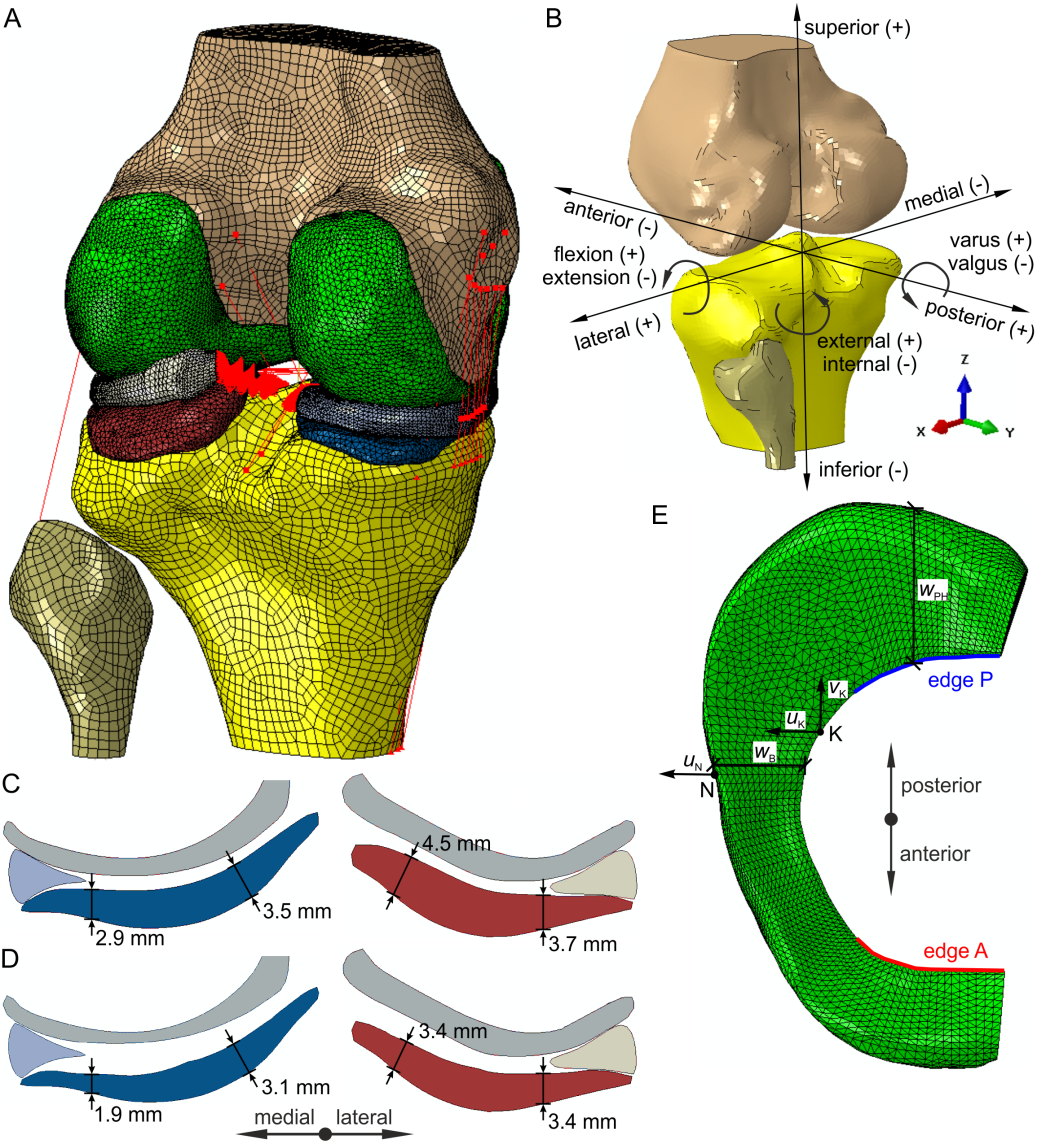
Finite element model of the knee joint. (A) Geometry and finite element mesh. (B) The directions of six degrees of freedom. (C) Geometry of the menisci and articular cartilage in the coronal plane for the intact model and model M. (D) Geometry of the menisci and articular cartilage in the coronal plane for the OA model and model G. (E) The definition of the medial meniscus edges A, P, widths *w*_B_, *w*_PH_ and translations *u*_K_, *v*_K_, *u*_N_.

The crucial factor in medial compartment knee osteoarthritis is the degradation of articulate cartilage (Wirth et al., 2010; Arno et al., 2012). In the present study alterations of medial knee OA geometry were introduced into model G and the OA model based on difference thickness maps from an MRI study (Favre et al., 2017) comparing the cartilage thickness of the KL 3 subgroup to the non-OA subgroup. The cartilage thickness was reduced in specific areas of the medial femoral condyle and both compartments of the tibial plateau, where significant differences were observed in the study (Favre et al., 2017). The cross-section of the menisci and articular cartilage in the coronal plane for models with intact geometry and models with altered geometry is presented in Figs. 1C and 1D, respectively. The continuous non-uniform offset of contact surfaces of the articular cartilage resulted in a decrease of 9.2 and 7% in the volume of tibial cartilage in the medial compartment and the lateral compartment, respectively.

### Materials

The Yeoh model was used to model an isotropic, hyperelastic, nearly incompressible behavior of the articular cartilage. The strain energy function for the Yeoh model has the following form

(1) \begin{eqnarray*}\Phi ={C}_{10}({\overline{I}}_{1}-3)+{C}_{20}({\overline{I}}_{1}-3)^{2}+{C}_{30}({\overline{I}}_{1}-3)^{3}+ \frac{1}{{D}_{1}} (J-1)^{2}+ \frac{1}{{D}_{2}} (J-1)^{4}+ \frac{1}{{D}_{3}} (J-1)^{6},\end{eqnarray*}

where ${\overline{I}}_{1}$ is the first invariant of the modified left Cauchy–Green tensor, *J* is the elastic volume ratio and *C*_10_, *C*_20_, *C*_30_, *D*_1_, *D*_2_, *D*_3_ are independent Yeoh material constants. The values of the parameters *C*_10_, *C*_20_ for normal and degenerated knee cartilage were taken from an experimental study (Robinson et al., 2016), see Table 1. In the paper, the constant *C*_30_ was assumed to be zero due to a lack of experimental data. The value of the parameter *D*_1_ was calculated based on the value of *C*_10_ and the value *v* = 0.45 of Poisson’s ratio (Table 1). Moreover, the last two terms in the Eq. (1) were neglected because a nonzero value of *D*_1_ was sufficient to model a nearly incompressible behavior of the articular cartilage.

**Table 1:** Material parameters of normal and osteoarthritic articular cartilage (Robinson et al., 2016).

cartilage	tibial	tibial OA	femoral	femoral OA
*C*_10_ (MPa)	2.0	1.1	1.4	1.2
*C*_20_ (MPa)	4.5	2.7	3.6	2.2
*D*_1_ (MPa^−1^)	0.0517	0.094	0.0739	0.0862

A linear, elastic and transversely isotropic material model was used for the menisci. The Young’s moduli in the circumferential direction *E*_*θ*_ and in the cross sectional plane *E*_*p*_, Poisson’s ratios *ν*_*θp*_, *ν*_*p*_ and the shear modulus *G* for healthy menisci were taken from papers (Lechner, Hull & Howell, 2000; Haut Donahue et al., 2003; Yang, Canavan & Nayeb-Hashemi, 2010), see Table 2. The material parameters of osteoarthritic menisci in models M and OA were assumed based on experimental data for the menisci with a grade 3 of degeneration (Fischenich et al., 2015). A significant decrease in the instantaneous compressive modulus was observed in both osteoarthritic menisci, therefore four times smaller Young’s modulus *E*_*p*_ was assumed in the axial and radial directions in which menisci are compressed (Fischenich et al., 2015; Fischenich et al., 2017), see Table 2. The unchanged Young’s modulus was used in the circumferential direction, because degenerated menisci retain a tensile modulus similar to that obtained for healthy menisci (Lechner, Hull & Howell, 2000; Fischenich et al., 2015). The same values of the Poisson’s ratios and the shear modulus were assumed in all models due to a lack of experimental study about effect of medial knee OA on these parameters.

**Table 2:** Material parameters assumed for normal and osteoarthritic menisci.

	*E*_*θ*_ (MPa)	*E*_*p*_ (MPa)	*G* (MPa)	*ν*_*θp*_	*ν*_*p*_
normal	120	20	57.7	0.3	0.2
osteoarthritic	120	5	57.7	0.3	0.2

A constitutive relation in the ligaments modelled by the axial connector elements was defined by relationship between the ligament tensile force *F* and its elongation *u*. The force in the ligaments is zero when the following condition is fulfilled:

(2) \begin{eqnarray*}F=0,\quad u\lt -{}_{r}{L}_{0},\end{eqnarray*}

where *ɛ*_*r*_ is the reference (initial) strain and *L*_0_ is the zero-length of a ligament bundle. The length *L*_0_ was computed based on the ligament length *L*_*r*_ and the strain *ɛ*_*r*_ in the reference configuration

(3) \begin{eqnarray*}{L}_{0}={L}_{r}/(1+{}_{r}).\end{eqnarray*}

For low values of elongation the relationship *F*(*u*) is nonlinear and given by

(4) \begin{eqnarray*}F= \frac{k(u+{}_{r}{L}_{0})^{2}}{4{L}_{0}^{2}{}_{l}} ,\quad -{}_{r}{L}_{0}\leq u\leq (2{}_{l}-{}_{r}){L}_{0},\end{eqnarray*}

where *k* is the ligament stiffness and *ɛ*_*l*_ is the transition strain, assumed as 0.03 (Butler, Kay & Stouffer, 1986). The values of *k* and *ɛ*_*r*_ are given for the whole ligaments and the specific ligament bundles in Table 3. For greater strains the ligament force is proportional to its elongation

(5) \begin{eqnarray*}F=k \left( \frac{u}{{L}_{0}} +{}_{r}-{}_{l} \right) ,\quad u\gt (2{}_{l}-{}_{r}){L}_{0}.\end{eqnarray*}

The meniscal horn attachments were modelled by nonlinear spring elements carrying only tensile forces. Insertion nodes for each meniscal attachment were identified on the basis of the MRI scans. The stiffness of each meniscal horn attachment was assumed based on the experimental linear stiffness, considering difference between its length in the FE models and an experimental study (Hauch, Villegas & Haut Donahue, 2010). The total tensile stiffness *k*_*h*_ and the spring stiffness *k*_*s*_ are reported for the meniscal horn attachments in Table 4.

**Table 3:** The stiffness parameters and the reference strains of the ligaments.

Ligament/ligament bundle	Stiffness *k*(N)	Reference strain *ɛ*_*r*_	Source
ACL - anteromedial	5800	0.06	Blankevoort et al. (1991), Butler et al. (1992), Grzelak et al. (2012)
ACL - posterolateral	3200	0.10	Blankevoort et al. (1991), Butler et al. (1992), Grzelak et al. (2012)
LCL	6000	0.05	Blankevoort et al. (1991), Yang, Canavan & Nayeb-Hashemi (2010)
MCL - anterior	2400	0.03	Gardiner, Weiss & Rosenberg (2001), Robinson, Bull & Amis (2005)
MCL - middle	2500	0.043	Gardiner, Weiss & Rosenberg (2001), Robinson, Bull & Amis (2005)
MCL - posterior	2500	0.05	Gardiner, Weiss & Rosenberg (2001), Robinson, Bull & Amis (2005)
MCL - deep	1300	0.03	Robinson, Bull & Amis (2005), Smith et al. (2016)
PCL - anterolateral	11400	−0.16	Race & Amis (1994), Moglo & Shirazi-Adl (2003)
PCL - posteromedial	2430	−0.03	Blankevoort et al. (1991), Race & Amis (1994)
AIML	750	0.00	Nelson & LaPrade (2000), Guess & Razu (2017)
ALL	750	−0.06	Kennedy et al. (2015), Drews et al. (2017)
POL	1700	0.05	Wijdicks et al. (2010)

**Table 4:** Parameters of meniscal horn attachments.

Meniscal horn	Lateral anterior	Lateral posterior	Medial anterior	Medial posterior
*l* (mm)	11.08	11.90	10.78	6.79
*k*_*h*_ (N/mm)	253.50	106.6	218.0	218.7
*N*	55	85	67	64
*k*_*s*_ (N/mm)	4.609	1.254	3.254	3.417

**Notes.**

*l*, the attachment length; *k*_*h*_, total tensile stiffness; *N*, number of nonlinear springs and *k*_*s*_, the spring stiffness.

The use of reduced material parameters for degenerated cartilage and menisci is the authors’ contribution to the description of material parameters for FEM models. The density for all soft tissues was assumed as 1,500 kg/m^3^ (Erdemir & Sibole, 2010) and for the bones as 2,000 kg/m^3^ (Maharaj, Maheswaran & Vasanthanathan, 2013).

### Finite element mesh

The menisci and the articular cartilage were discretized by the 4-node tetrahedral elements (C3D4) with linear shape functions. The rigid shell triangular (R3D3) and quadrilateral (R3D4) elements were used to model the bones. The average size of 0.8 mm, 1.1 mm and 1.2 mm was assumed for the menisci, the tibial cartilage and the femoral cartilage, respectively. The intact knee model consisted of the total number of 69,808 nodes and 267,479 finite elements. The mesh convergence test was performed to verify the correctness of FE mesh. A refined discretization (319,274 nodes) was generated by reduction of finite element size by a factor of two in the menisci and articular cartilage. The differences in contact pressures and hoop stresses between the refined discretization and the one used in the paper were less than 4%. Thus, the assumed FE mesh was considered adequate for numerical simulations (Zienkiewicz & Taylor, 2002).

### Finite element analysis

Only stance phase of the gait cycle was simulated in the FE code Abaqus, because it has been well documented *in-vivo* (Gilbert et al., 2014; Bergmann et al., 2014) and through modelling (Shriram et al., 2019) that tibiofemoral contact forces and stresses are small during the swing phase compared to the stance phase. In the initial static step the equilibrium state between contacting surfaces was obtained for the imposed reference strains in the ligaments (Table 3). Then the initial loads from the gait cycle were applied to the model within one second in a dynamic step. The dynamic implicit solver was used in the nonlinear FE analysis of the stance phase that accounts for 60% of the duration of the gait cycle of 1.0674 s (Bergmann et al., 2014).

### Boundary conditions and loads

The femur, tibia and fibula were modelled as rigid bodies due to significantly greater stiffness of bones than soft tissues. Kinematics of the bones is defined by the six degrees of freedom of their so-called reference points (RPs). The tibia RP was located on the intersection of the tibial mechanical axis and the surface of the tibial plateau. The femur RP was assumed at the midpoint between the medial and lateral epicondyles. The orthogonal joint coordinate system was defined based on the tibial mechanical axis and the femur flexion axis. The positive directions for translational and rotational degrees of freedom in the knee joint are presented in Fig. 1B.

**Figure 2:**
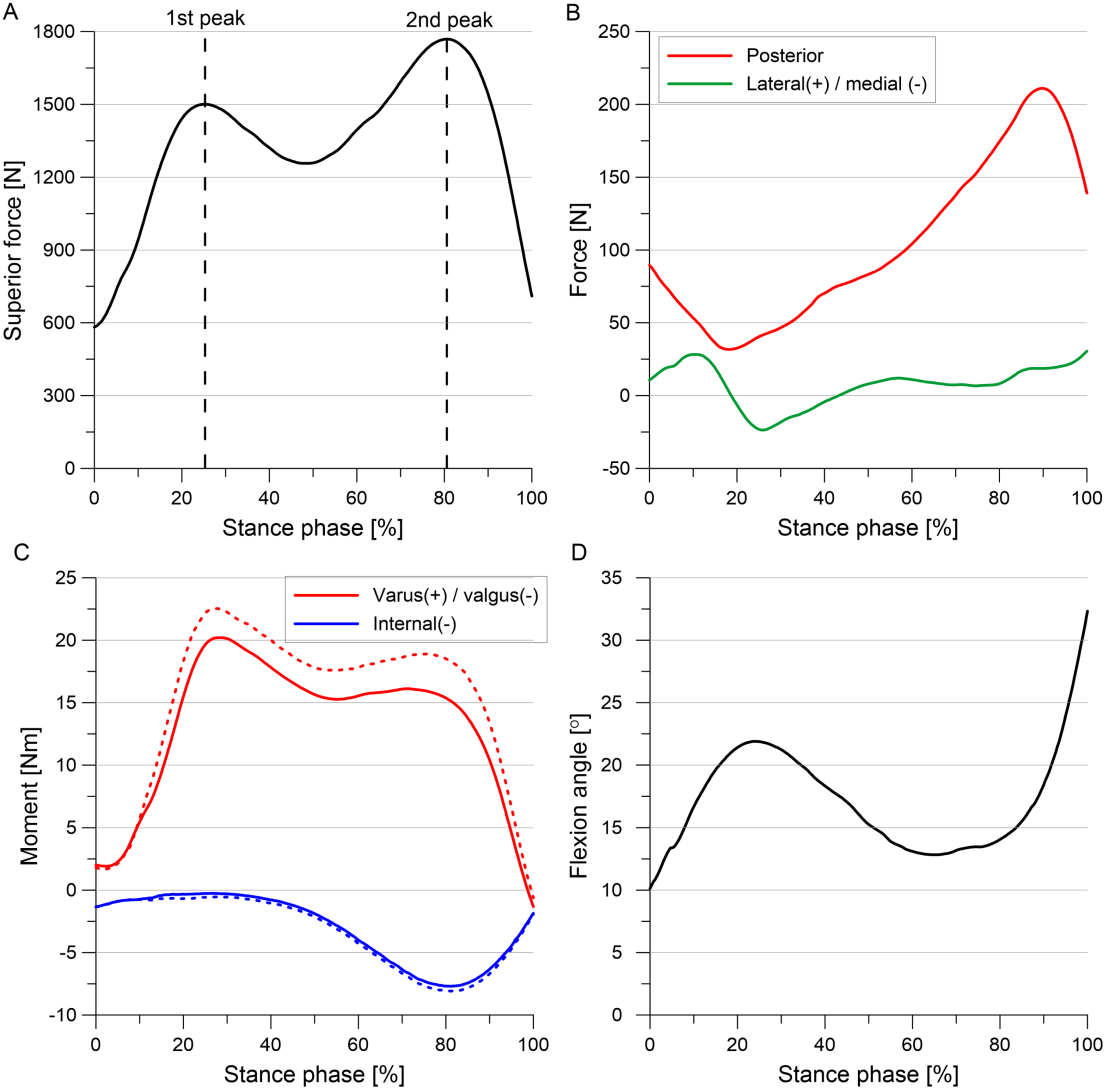
External loads and moments applied to the tibia during the stance phase of the gait cycle. (A) Superior force. (B) Posterior force and lateral(+) / medial(-) force. (C) Internal torque and varus(+) / valgus(-) torque. (D) Flexion angle. The corrected moments applied to models G and OA are denoted by dashed line.

Forces and moments acting in the knee during the gait cycle were taken from experimental studies (Bergmann, 2008; Bergmann et al., 2014), because gait data was not available for the analyzed knee. First, the standardized average loads (file Walking_AllLoadLevels from database OrthoLoad (Bergmann, 2008)) were multiplied by 68/75 to re-calculate them from an average body weight of 75 kg to the volunteer weight of 68 kg. Then the forces and moments were transformed from the implant-based system to the knee joint coordinate system. The obtained loads (Fig. 2) were applied to the tibia RP and they followed rotations of the tibia RP during the gait simulation.

The greater reduction in thickness of articular cartilage in the medial compartment resulted in approximately 1° greater varus rotation in models G and OA than in the intact model in gait simulations. It is well established that the greater varus alignment causes an increase in knee varus moment in patients with knee OA (Moyer et al., 2010; Sharma, 2007). However, our model of the knee joint cannot simulate an increase in varus moment as the effect of medial movement of the mechanical axis of the leg with respect to the knee. Therefore, corrected varus moment (denoted by dashed line in Fig. 2) computed for 1° greater varus alignment was used in models G and OA to directly take into account this effect. The loading cycles from 7 subjects (files K1L, K2L, K3R, K5R, K7L, K8L, K9L from the database OrthoLoad (Bergmann, 2008)) were averaged intra-individually using basic average method (Bergmann et al., 2014) to determine corrected standardized average varus moment for the volunteer weight of 68 kg. Load data for K6L subject with valgus alignment (from the database OrthoLoad (Bergmann, 2008)) was not used in these computations to obtain the increased varus moment for 1° greater varus alignment than in the intact model. The transformation of the corrected varus moment to the knee joint coordinate system resulted in a slight change in the internal moment, see Fig. 2.

All degrees of freedom (DOFs) were fixed at the femur RP. During the gait simulation five degrees of freedom were released at the tibia RP, except flexion rotation which was prescribed (see Fig. 2D). The hard contact approach was used to model interaction between external surfaces of the articular cartilage and menisci. A friction with the very low friction coefficient of 0.02 was assumed between the contacting surfaces based on a previous study (Warnecke et al., 2019). Moreover, an automatic stabilization factor of 0.2 was used to improve the convergence of computations in the initial step. A “tie” constraint was defined between the bone RPs and cartilage surfaces to enforce the rigid connection at the cartilage-bone interfaces. The fibula RP was constrained with the tibia RP using a “kinematic coupling” option.

### Model validation

The FE models were validated against the expected results from literature. First, the passive knee flexion test up to 70 degrees was performed for the intact model and the OA model to verify correctness of the tibiofemoral kinematics. The tibial kinematics with respect to the femur predicted by the FE models were compared with the experimental tibiofemoral kinematics measured in 23 cadaveric lower limbs (Germain et al., 2016). Maximal tibial contact pressures obtained in the intact model for the first and second peaks of total force were verified against the experimental values measured with pressure sensors in a study (Bedi et al., 2010). Moreover, the change in location of the peak contact pressure between the first peak and the second peak of total force observed in the intact model was compared with the results of experimental studies (Bedi et al., 2010; Gilbert et al., 2014). The absolute difference in width of the medial meniscus midbody between the OA model and the intact model obtained for the first peak was verified against the corresponding difference measured in a MRI study (Wenger et al., 2013) between the subjects with knee OA and reference subjects. Moreover, the medial translation of the medial meniscus at point N computed in the intact model was compared with values of meniscal extrusion determined in studies (Zhang et al., 2019; Li et al., 2020).

### Outcome variables

Outcome variables were presented in the results section for the first and second peaks of total force corresponding to 25% and 81%, respectively, of the stance phase of the gait cycle (Fig. 2A). The total force was defined as the sum of the resultant forces in the medial and lateral compartments of the tibiofemoral joint. The von Mises stresses and hoop stresses were analyzed for the medial meniscus to investigate the location of potential meniscal tear initiation. The von Mises stress is the equivalent stress that allows the comparison of three dimensional stress state with the uniaxial yield stress. The hoop stresses are tensile stresses along the circumferential collagen fibers of menisci that are generated by axial forces in the knee joint. The hoop stresses may be compared with maximum tensile stress determined for the circumferential specimens. Strains were not reported in the results section, because they are proportional to stresses in the menisci. The meniscal deformation and tibiofemoral kinematics were presented to assess the influence of medial knee OA on the biomechanics of the medial meniscus. Curves for medial/lateral translation were not given because it was low in all models.

The hoop stresses and translations of the medial meniscus were measured at specific points and edges shown in Fig. 1E. The average and maximal hoop stresses were determined on the inner edge A of the anterior horn and on the inner edge P of the posterior horn. The meniscal translations were computed relative to the tibia. The posterior meniscal translation *v*_K_ was measured at point K and the translations *u*_K_ and *u*_N_ in the medial direction at points K and N, respectively. Point K was chosen on the inner edge of the medial meniscus body as the closest point to the location of the maximal hoop stress in the OA model. Point N was selected on the outer edge of the medial meniscus body in the location of maximal medial translation. Moreover, medial translation at point N is close to a meniscal extrusion of the medial meniscus body. The changes in medial meniscal width Δ*w*_B_ and Δ*w*_PH_ were calculated in the meniscal midbody and the posterior horn, respectively.

The relative and absolute changes in values of outcome variables for models G, M and OA were computed relative to the values obtained for the intact knee model. The maximal contact pressure between articular cartilage, the maximal von Mises stress and hoop stress in the medial meniscus, medial translation and change of the medial meniscus width, the tibial translations and rotations were compared between the intact model and the OA model to assess the influence of osteoarthritis on the biomechanics of the medial meniscus. Moreover, the differences in contribution of the medial meniscus in carrying of compressive load, the maximal hoop stress, posterior and medial translation of the medial meniscus were compared between models G and M to evaluate the influence of changes in geometry and material parameters on the specific results, respectively.

## Results

### Stress state

**Figure 3:**
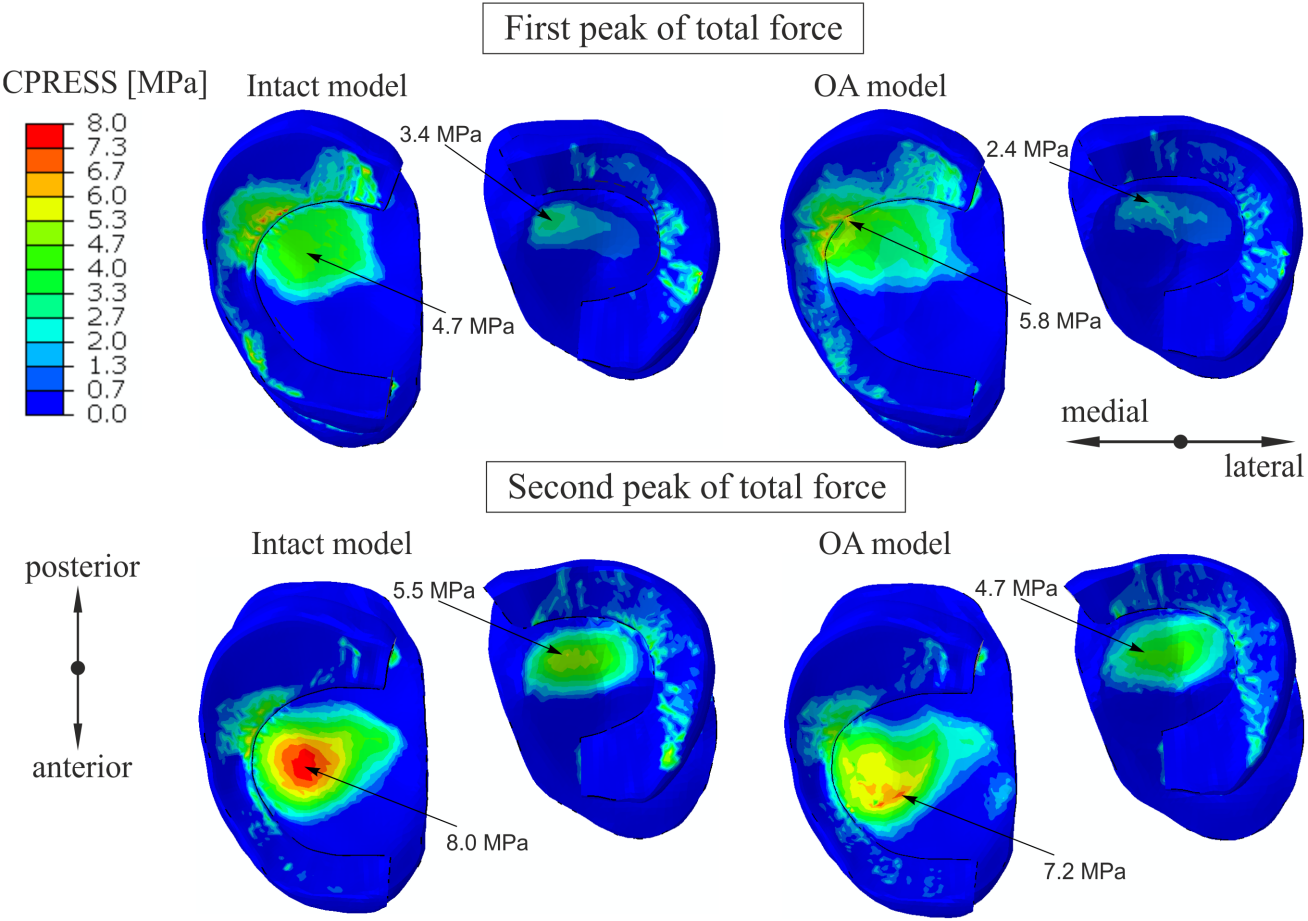
The contour plots of the contact pressure on the tibial articular cartilage and menisci surfaces. The plots were obtained for the intact knee model and the OA model at 25% (first peak) and at 81% (second peak) of the stance phase.

Similar distribution of contact pressure on the surfaces of the tibial cartilage and menisci was observed in the intact knee model and the OA model for the first peak in Fig. 3. A 23% increase and a 29% decrease in the maximal value of the contact pressure between the articular cartilage was obtained in the medial and lateral compartment, respectively. The contour plots for the second peak (Fig. 3) show a 10% decrease in maximal contact pressure and anterior shift of its location on the medial tibial cartilage in the OA model. These changes are the effect of the greatest reduction in cartilage thickness at the central part of the medial tibial cartilage (Favre et al., 2017).

**Figure 4:**
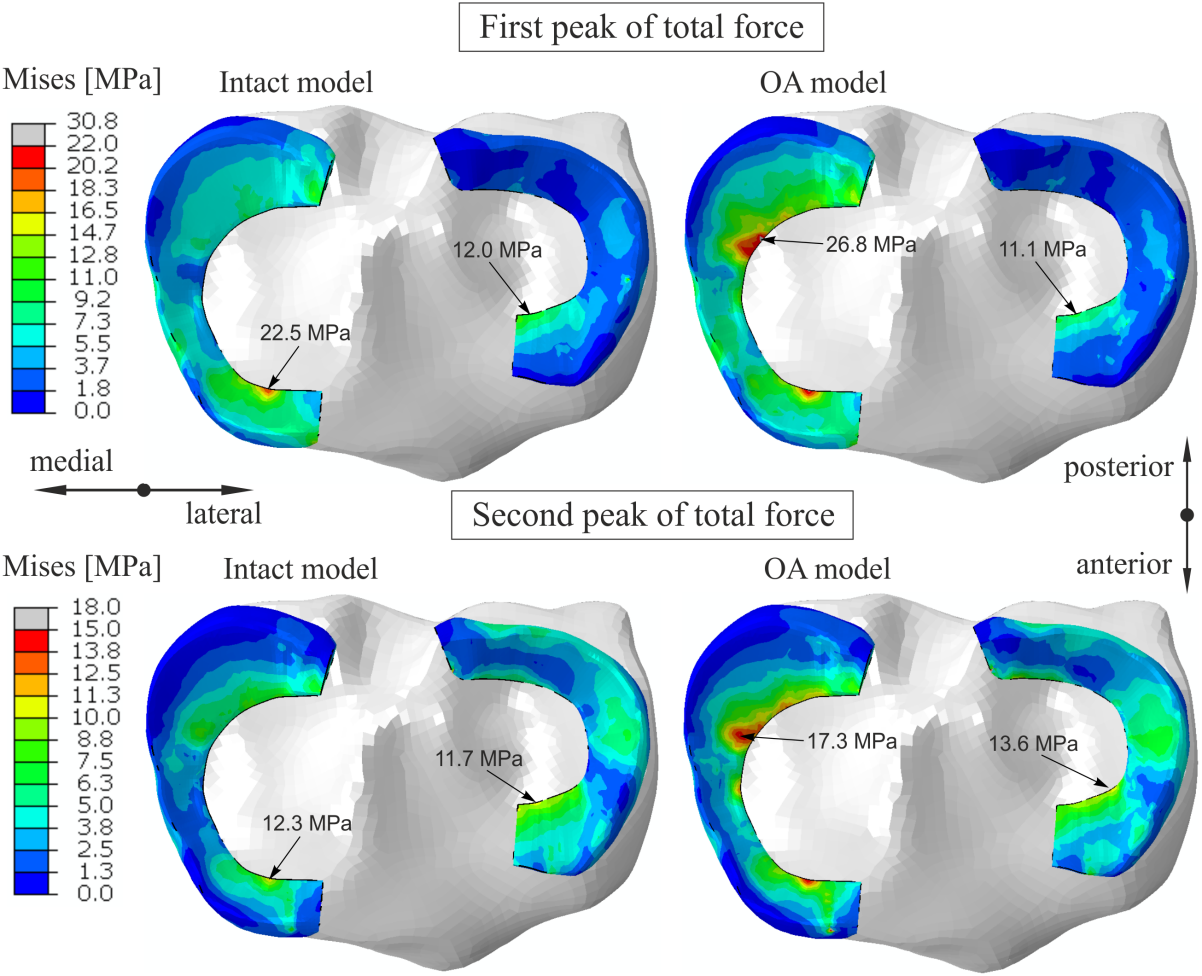
The contour plots of the von Mises stress on the proximal surfaces of the menisci. The plots were obtained for the intact knee model and the OA model at 25% (first peak) and at 81% (second peak) of the stance phase.

The contribution of the medial meniscus in carrying of compressive load increased from 48% to 53.7 in model G and decreased to 44.5% in model M for the first peak (see Table 5). Consequently, a minor reduction in forces between the tibial cartilage and the menisci was obtained in the OA model. The maximum von Mises stress of 22.5 MPa for the first peak was obtained in the anterior horn of the medial meniscus body in the intact model (Fig. 4). While, the maximum von Mises stress of 26.8 MPa was observed in the posterior part of the medial meniscus body in the OA model.

**Table 5:** The contribution of menisci and articular cartilage in carrying of compressive load for different knee models at 25% of the stance phase (first peak).

	Force between articular cartilage		Force between tibial cartilage and meniscus			
Compartment	lateral	medial	lateral		medial	
	[N]	[N]	[N]	[-]*	[N]	[-]*
Intact model	179.01	687.61	176.94	49.7%	634.84	48.0%
Model G	102.69	598.4	200.6	66.1%	694.58	53.7%
Model M	218.64	720.77	142.52	39.5%	578.33	44.5%
OA model	144.18	659.60	158.95	52.4%	617.32	48.3%

**Notes.**

*Percentage values are computed relative to total force value in a given knee compartment.

**Figure 5:**
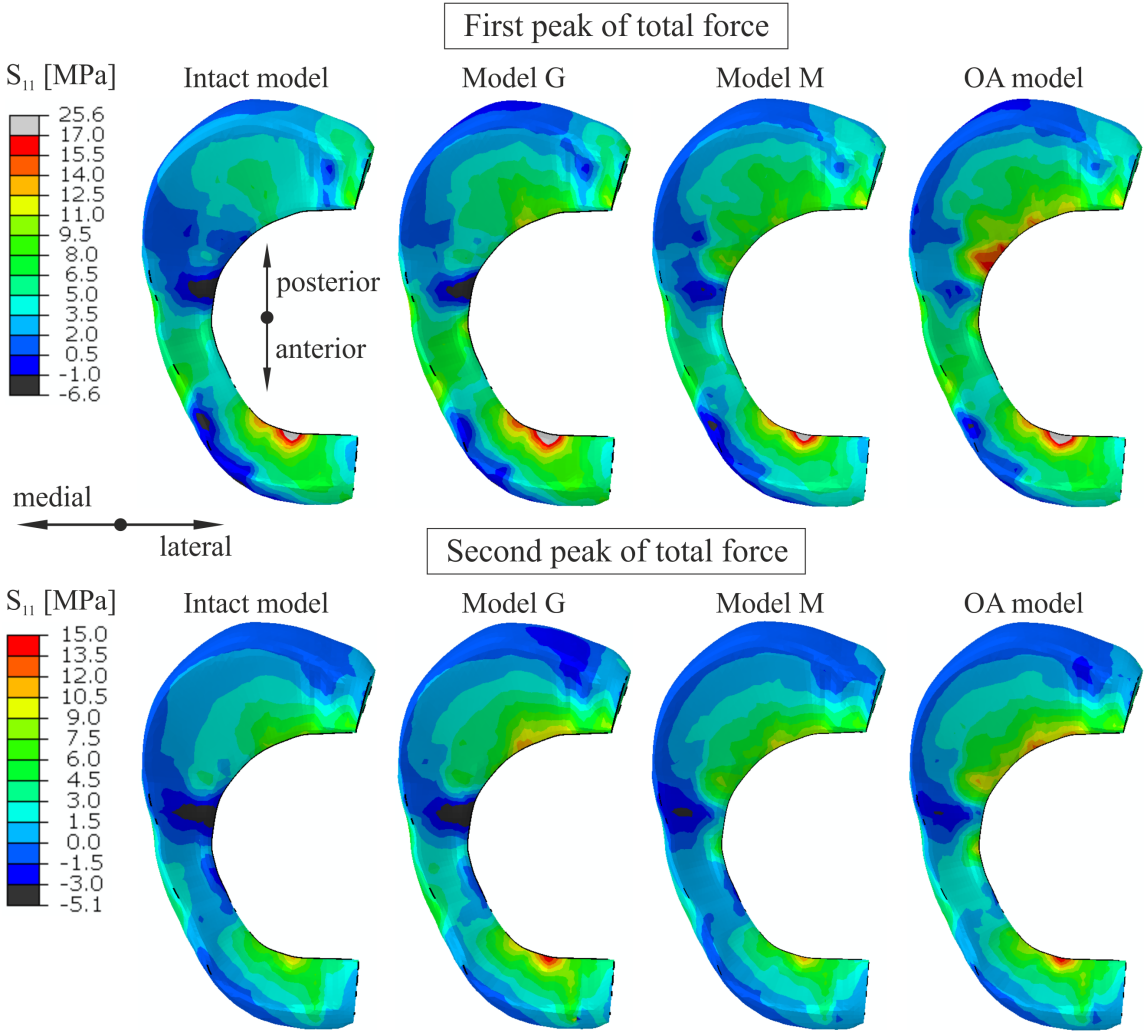
The contour plots of the hoop stress on the proximal surface of the medial meniscus. The plots were obtained for the different knee models at 25% (first peak) and at 81% (second peak) of the stance phase.

**Figure 6:**
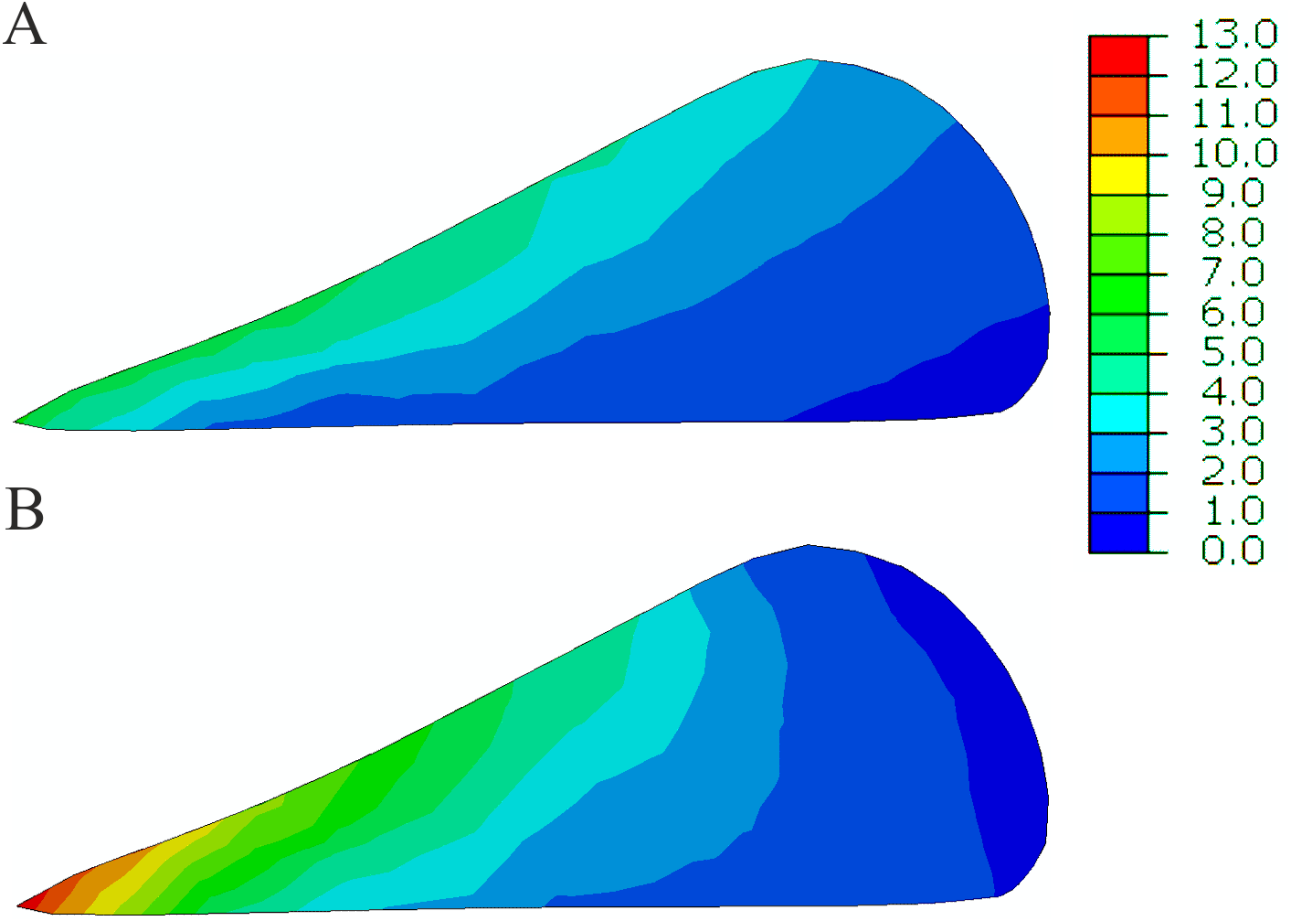
The contour plots of the hoop stress (MPa) in the sagittal cross section of the posterior horn of the medial meniscus at 25% of the stance phase. (A) The intact knee model. (B) The OA model.

The maximal and average hoop stresses in the medial meniscus are compared in Fig. 5 and Table 6. The maximal hoop stress was 83% greater for the first peak and 58% greater for the second peak on edge P (see Fig. 1E) in the OA model compared to the intact model. Similar values of hoop stress were obtained for all models in the anterior horn of the medial meniscus for the first peak. Table 6 and Fig. 5 show that reduction in articular cartilage thickness (model G) caused an increase of approximately 70% in the maximal value and an increase of 100% in the average value of hoop stress on edge P for the first peak. Decrease in compressive stiffness (model M) resulted in lower maximal and average hoop stresses on both edges than in model G (Table 6). However, a considerably greater increase in hoop stress from 2.6 MPa to 12.1 MPa was observed near point K in model M than in model G (6.64 MPa) for the first peak. Approximately seven times higher hoop stress was obtained near point K in the OA model (Table 6). A substantial change in the distribution of hoop stress was observed in the sagittal cross section of the posterior horn of the medial meniscus in the OA model, see Fig. 6.

**Table 6:** The comparison of maximal and average hoop stresses, medial meniscus deformation and tibia varus rotation for different knee models at first peak and at second peak of the stance phase.

		First peak of total force				Second peak of total force			
		Intact model	Model G	Model M	OA model	Intact model	Model G	Model M	OA model
Maximal hoop stress (MPa)	Posterior horn	8.62	14.38	12.72	15.78	9.23	13.38	11.86	14.62
	Anterior horn	22.76	24.75	22.69	25.32	12.45	16.92	13.24	17.08
	Point K	2.56	6.64	12.10	18.33	2.55	5.41	7.86	11.28
Average hoop stress (MPa)	Posterior horn	3.94	7.97	7.37	9.98	6.04	8.76	7.23	9.07
	Anterior horn	11.64	12.84	11.33	12.79	6.30	8.77	6.37	8.42
Medial translation	*u*_N_ (mm)	1.92	3.01	1.54	2.39	2.72	3.36	2.55	3.03
	*u*_K_ (mm)	2.35	3.58	2.80	4.08	2.96	3.59	3.40	3.99
Posterior translation	*v*_K_ (mm)	4.44	3.07	5.77	4.65	−0.56	−1.16	−0.32	−0.80
Change of meniscal width	Δ*w*_B_ (mm)	−0.45	−0.76	−1.13	−1.88	−0.47	−0.62	−1.08	−1.32
	Δ*w*_PH_ (mm)	−0.48	−0.22	−1.34	−0.86	0.05	0.16	−0.02	0.34
Varus rotation (°)		0.81	2.29	0.84	2.15	0.61	1.48	0.43	1.27

**Notes.**

The stresses were determined on the inner edges of the medial meniscus: A (anterior horn), P (posterior horn). The maximal value of hoop stress from finite elements with node K was reported. The medial translations *u*_*K*_, *u*_*N*_ and posterior translation *v*_*K*_ were computed relative to the tibia at points K and N of the medial meniscus (see Fig. 1C). Δ*w*_B_ - change of medial meniscal midbody width and Δ*w*_PH_ - change of medial meniscal posterior horn width.

### Meniscal deformation

**Figure 7:**
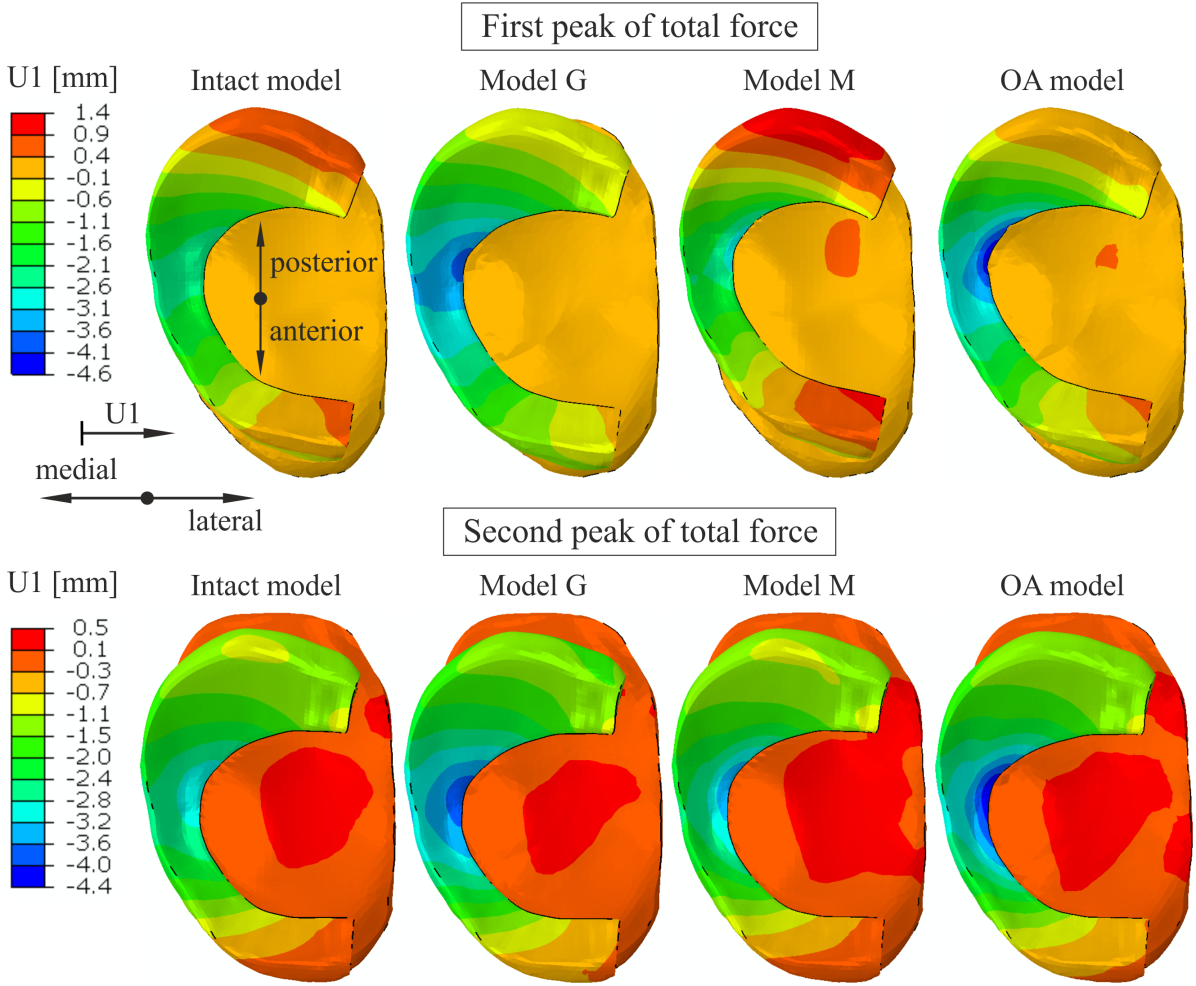
The contour plots of lateral(+)/medial(-) translation measured relative to the tibia on the proximal surface of the medial meniscus. The plots were obtained for the different knee models at 25% (first peak) and at 81% (second peak) of the stance phase.

An increase of 74% and 25% in medial(-) translation of the medial meniscus was obtained at point K and point N (compare Fig. 1E), respectively, in the OA model for the first peak (see Table 6). The greatest increase of 57% in the medial translation at point N was determined in model G. Considerably greater deformation in the medial direction was observed in the inner part of the medial meniscus body (Fig. 7) in models M and OA due to the reduction of the meniscal body width *w*_B_ by 1.13 mm and 1.88 mm, respectively. A decrease in width *w*_B_ was approximately 4 times greater in the OA model compared to the intact model (Table 6). The greatest reduction of the medial meniscal posterior horn width by 1.34 mm was observed in model M. The posterior translation at point K *v*_K_ decreased by 31% in model G and increased by 30% in model M (Table 6). The similar values of posterior translation *v*_K_ were observed in the intact model and the OA model. The differences in meniscal deformation between the models are generally lower for the second peak than for the first peak (Table 6).

### Knee kinematics

**Figure 8:**
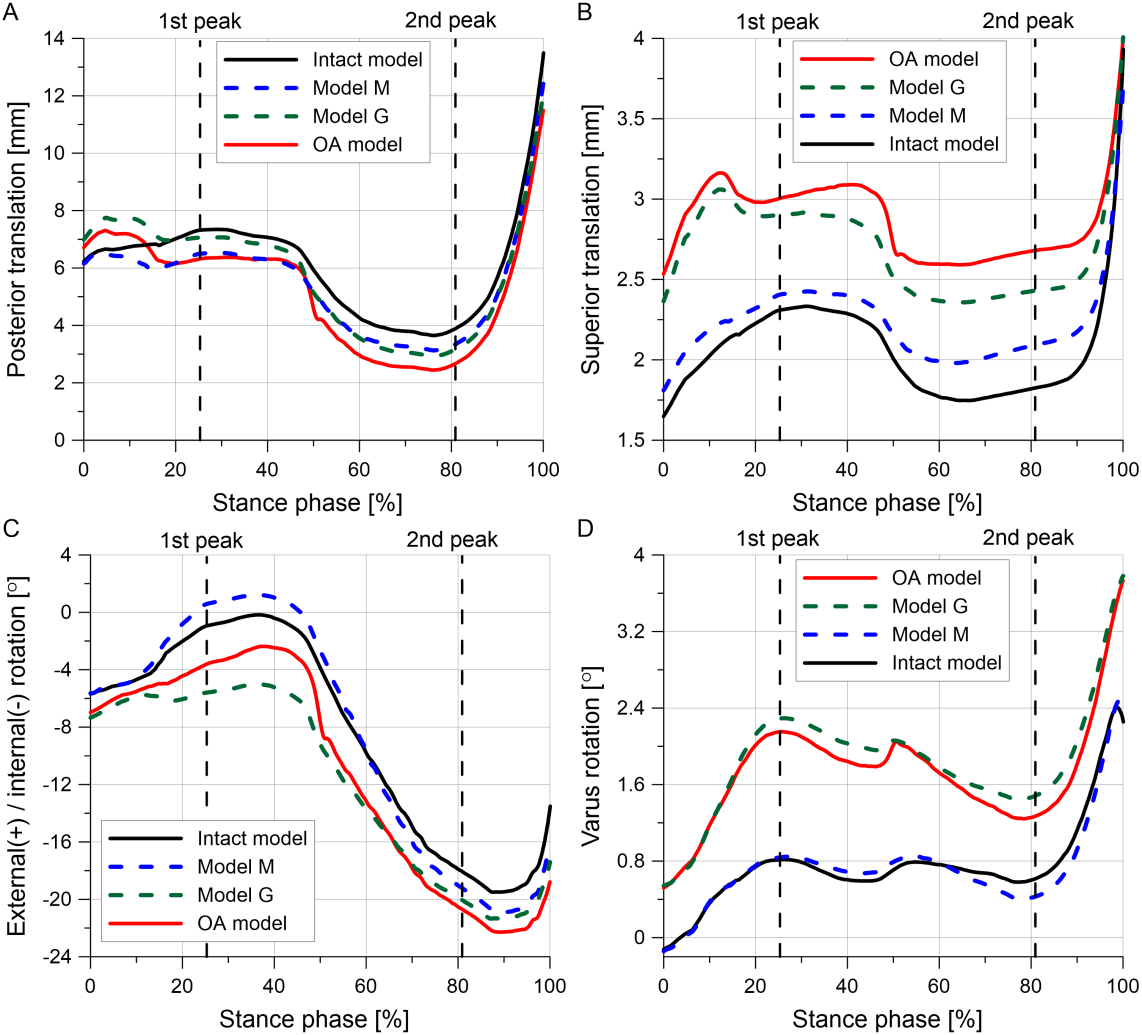
Tibial kinematics relative to the femur during the stance phase of the gait cycle for different knee models. (A) Posterior translation. (B) Superior translation. (C) External(+)/internal(-) rotation. (D) Varus rotation.

The tibial translations and rotations during the stance phase of the gait cycle are presented in Fig. 8. The posterior translation of the tibia is smaller for almost the entire stance phase in the OA model than in the intact model (Fig. 8A). The reduction in articular cartilage thickness caused an increase in superior translation, internal rotation and varus rotation in models G and OA (Figs. 8B–8D). The greater increase in the varus rotation in the OA model was observed for the first peak (1.34°) than for the second peak (0.66°) (see Table 6).

### Validation

**Figure 9:**
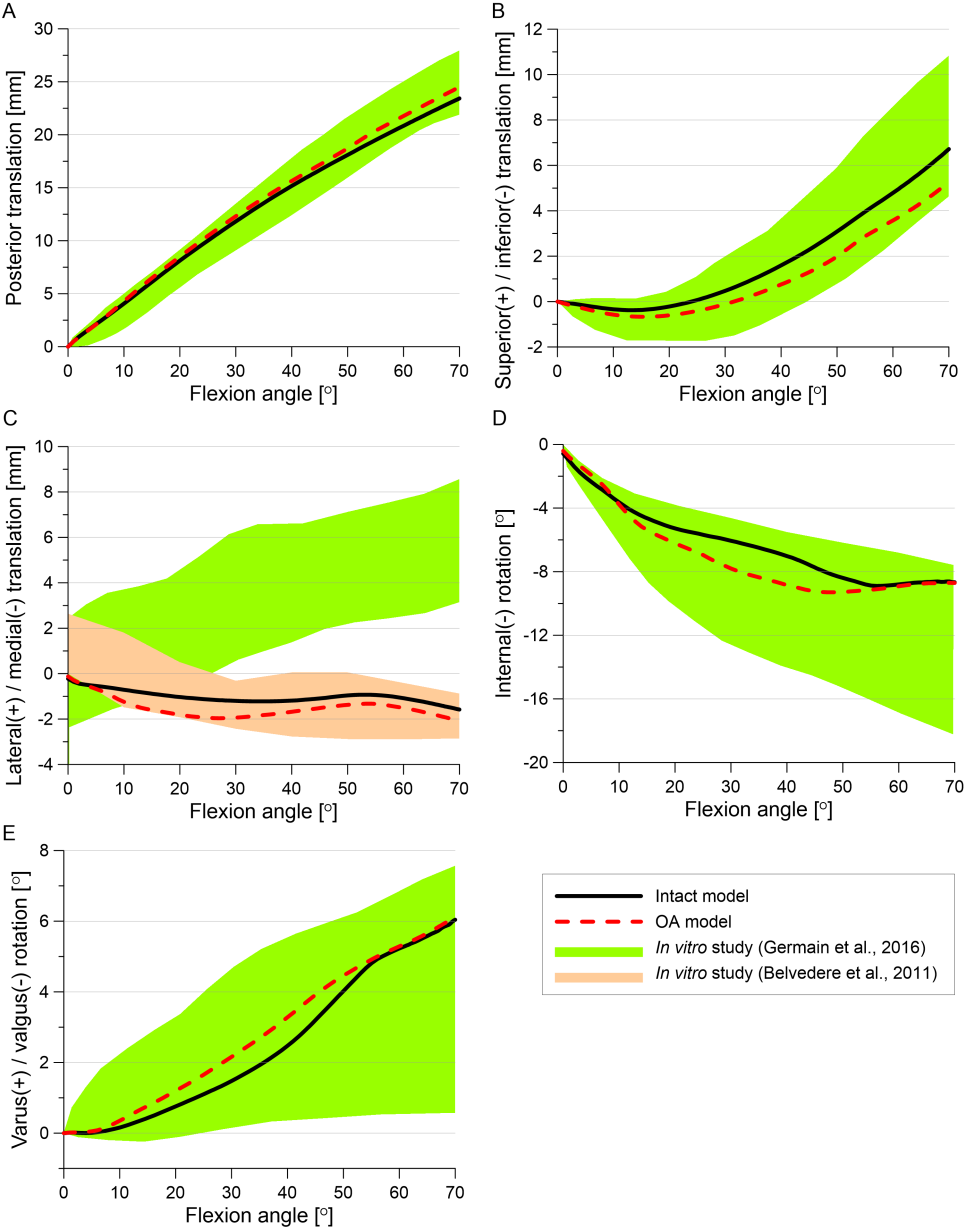
A comparison of tibial kinematics relative to the femur obtained in the intact model and the OA model with experimental range of motion during the passive knee flexion test.

The tibial translations and rotations obtained for the intact model and the OA model in the passive knee flexion simulation (see Fig. 9) were within range measured in the physical experiment (Germain et al., 2016). Only the medial translation of the tibia relative to the femur obtained in our study was beyond the physiological corridor from the previous study (Germain et al., 2016), but it was in the experimental range from an *in vitro* study (Belvedere et al., 2011). Moreover, tibial translation in the medial direction was reported in the majority of studies (see *i.e.,* Li et al., 2007; Seisler & Sheehan, 2007; Belvedere et al., 2011).

Maximal tibial contact pressures of 5.9 MPa and 8 MPa obtained in the intact model for the first and second peaks, respectively, are consistent with values of 6 ± 0.5 MPa and 7.4 ± 0.6 MPa from the experimental study (Bedi et al., 2010). The anterior shift of the maximal contact pressure location on the medial tibial cartilage between the first peak and the second peak of total force (Fig. 3) was also observed in the previous studies (Bedi et al., 2010; Gilbert et al., 2014).

The medial meniscal width was 1.43 mm narrower in the OA model than in the intact model for the first peak, see Table 6. This value is in good agreement with absolute difference of 1.39 mm in meniscal width reported in the study (Wenger et al., 2013). The values of 1.92 mm and 2.72 mm of translation *u*_N_ for the first peak and second peaks, respectively, are consistent with values of 1.8 mm and 3.16 mm of the medial meniscus extrusion from the studies (Zhang et al., 2019; Li et al., 2020).

## Discussion

In the present study the stress state and deformation of the medial meniscus in medial knee OA was investigated during the stance phase of gait. We analyzed the influence of 8% loss of cartilage volume corresponding to KL 3 and, for the first time, the effect of a decrease in the material parameters of the cartilage and menisci. We confirmed that the tibiofemoral kinematics, the maximal tibial pressure and deformation of the medial meniscus determined in the numerical simulations were consistent with the experimental results from literature. Arno et al. (2012) suggested that the meniscus is not able to reduce forces and pressures acting between the articular cartilage and prevent degeneration in medial knee OA. In our previous study (Łuczkiewicz et al., 2016) we showed that the reduction in the role of the meniscus in the transmission of static load in the medial compartment may be attributed to incongruence between contact surfaces resulting from a 50% reduction in cartilage thickness. Here, the decrease in force between the tibial cartilage and the medial meniscus in model M (Table 5) showed that the medial meniscus did not take a greater load in the OA model due to the reduction in its compressive stiffness.

Considerable changes in distribution of the von Mises stress (Fig. 4) between the intact model and the OA model were observed at the posterior part of the medial meniscus body and on the inner edge P of the posterior horn. These differences were mainly caused by increase in hoop stress, because changes in other stress components were small. The maximal von Mises stress and the sevenfold increase in the hoop stress near point K in the OA model suggest that the changes in geometry and material parameters characteristic of medial knee OA may contribute to the formation of radial tears in the middle segment of the medial meniscus. This type of meniscal tear is the most common in patients with medial knee OA, and a distinctive symptom is pain while walking (Kamimura et al., 2015). The considerable increase in the maximal value of hoop stress on the inner edge P of the medial meniscus in the OA model may cause initiation of posterior root tears. A flap tear of the middle or posterior segment of the medial meniscus may also be the result of the higher stress levels associated with medial knee OA. All of these three types of meniscal tears were arthroscopically observed in 86% of knees in a one study (Kamimura et al., 2015) and in 71% of knees in another study (Kamimura et al., 2019).

The greater reduction in cartilage thickness in the medial compartment caused asymmetric joint space narrowing (Fig. 8B) and an increase in the tibial varus rotation (Fig. 8D) in models G and OA. The considerably greater medial shift of the medial meniscus midbody in model G than in the intact model (Fig. 7) is the effect of the greatest reduction in the joint space between the bones in this region. The increased medial translation of the middle segment of the meniscus in models G and OA resulted in greater stretching of both horns of the medial meniscus for the second peak of total force (Table 6). For the first peak, the largest medial deformation was observed in the posterior part of the meniscus body (Fig. 7); therefore, an increase in the hoop stress was substantially greater in the posterior horn than in the anterior horn (Table 6). The medial shift of the meniscus body caused bending of the meniscal posterior horn in the axial plane. Figure 6 shows that the increased bending changed the location of the maximal hoop stresses in the sagittal cross section from the top edge of the meniscus in the intact model to the meniscus corner at the inner edge P in the OA model. Such stress redistribution resulted in a considerable increase in peak hoop stress in the posterior horn of the medial meniscus for a relatively small increase in total tensile force.

Reduction of the material parameters of the cartilage and menisci in model M had a small influence on tibial kinematics (Fig. 8). The medial translation of the medial meniscus body and the hoop stresses on edges A and P were also lower in model M than in model G (Table 6). However, a greater increase in hoop stresses at the posterior part of the medial meniscus body was observed in model M compared to model G. This suggests that the loss of mechanical properties by the menisci and cartilage may have a greater influence on the initiation of radial tears of the meniscal middle segment than the reduction in cartilage thickness. Almost a fivefold increase in the hoop stresses near point K in model M for the first peak was caused by large deformation in the radial direction at the internal part of the meniscal midbody. This local deformation is attributed to a decrease in the meniscal width, Δ*w*_B_ (Table 6), resulting from high contact pressure near point K and fourfold lower meniscal compressive moduli in the axial and radial directions.

It is well established that meniscal lesions may lead to knee OA due to reduced congruence of the knee joint and greater stresses in the articular cartilage. However, knee osteoarthritis could also lead to meniscal tears because of the degenerative changes and weakening of meniscal structure (Englund, Guermazi & Lohmander, 2009). The degeneration process may result in decreased tensile strength of the medial meniscus and, consequently, an increased risk of meniscus damage for the normal knee joint loads. Our results showed that the degenerative changes in medial knee OA caused the greater meniscal deformation and an increase in the stresses on the inner edges of the medial meniscus. High stress level is a risk factor for initiation of meniscal tear, which in turn could accelerate the knee OA process. Thus, the results of this study may help to explain the causes for high prevalence of the meniscal lesions among patients with knee osteoarthritis.

The influence of medial knee OA on meniscal extrusion has been reported in previous studies (Arno et al., 2012; Wenger et al., 2013; Łuczkiewicz et al., 2016). The effect of a change in the meniscus cross-sectional shape on meniscal extrusion has been previously discussed (Łuczkiewicz et al., 2015; Luczkiewicz et al., 2018). In our model, the medial meniscal body extrusion estimated by the translation *u*_N_ at point N was the greatest in model G (Table 6), which confirms that the geometry of articular cartilage is the most important factor influencing meniscal extrusion. We obtained an increase in medial meniscal extrusion in the medial knee OA model, in agreement with previous works (Wenger et al., 2013; Łuczkiewicz et al., 2016).

This study has some limitations. The knee capsule and muscles were not taken into account in the FE model which may slightly affect tibial kinematics. This limitation has minor influence on the results obtained for the medial meniscus, because we applied the forces and moments measured in the tibiofemoral joint and created the detailed FE model which includes MTL, MFL and AIML ligaments attached to the medial meniscus. The single-phase hyperelastic Yeoh material model was used for the articular cartilage. However, the incompressible material model is a practical substitute in three-dimensional modeling of the short-time biphasic response of soft tissues (Ateshian, Ellis & Weiss, 2007). The same value of the shear modulus was assumed in all knee models due to a lack of experimental data on this parameter for medial knee OA. The biomechanical degradation in OA knee joint due to inflammation was not considered in the study. The generic flexion data and standardized loads were used in the FEM analyses which is justified, because the study is not clinical in nature.

## Conclusions

In summary, the maximal von Mises stress and nearly sevenfold increase in the hoop stresses were obtained in the posterior part of the medial meniscus body in the model of medial knee OA. Higher stress levels in the OA model than in the intact model were associated with a greater medial shift of the meniscus body, resulting from a reduction in the thickness of degenerated articular cartilage and about four-fold greater decrease in meniscal body width mainly due to the lower compressive stiffness of the menisci. The changes in articular cartilage geometry had greater influence on the knee joint kinematics and stresses in both horns of the medial meniscus, while the loss of mechanical properties by the soft tissues resulted in greater hoop stresses in the posterior part of the medial meniscus body. The considerable increase in hoop stresses demonstrated that changes in both geometry and material parameters characteristic of medial knee OA may contribute to the initiation of radial tears in the middle segment and the posterior horn of the medial meniscus.

## Additional Information and Declarations

### Data Availability

The following information was supplied regarding data availability: The text files containing all information about 3D models and ready for computations are available in the University repository: Intact model:
https://mostwiedzy.pl/pl/open-research-data/3d-intact-knee-model-used-in-analysis-of-the-medial-meniscus-biomechanics-in-the-osteoarthritic-knee,526044435535994-0?_share=ad04a98c9c69e33f
Model G:
https://mostwiedzy.pl/pl/open-research-data/3d-knee-model-g-with-reduced-thickness-of-articular-cartilage-input-text-file-for-computation,526045649549643-0?_share=978275e89c9f3202
Model M:
https://mostwiedzy.pl/pl/open-research-data/3d-knee-model-m-with-decreased-material-parameters-of-the-cartilage-and-menisci-input-text-file-for-,52605090757330-0?_share=949b4f783cd412ff
OA model:
https://mostwiedzy.pl/pl/open-research-data/3d-model-of-osteoarthritic-oa-knee-joint-for-analysis-of-the-medial-meniscus-biomechanics-input-text,526051623523702-0?_share=d96c964f3c2f7635.
